# The mesenteric defects in laparoscopic Roux-en-Y gastric bypass: 5 years follow-up of non-closure versus closure using the stapler technique

**DOI:** 10.1007/s00464-017-5415-2

**Published:** 2017-02-15

**Authors:** Ebrahim Aghajani, Bent J. Nergaard, Bjorn G. Leifson, Jan Hedenbro, Hjortur Gislason

**Affiliations:** Department of Surgery, Aleris Hospital, Fredrik Stangs gate 11–13, 0264 Oslo, Norway

**Keywords:** Bariatric surgery, Laparoscopic Roux-en-Y gastric bypass, Internal hernia, Mesenteric defects closure, Complication, Peterson

## Abstract

**Background:**

Internal hernia (IH) is a common complication of laparoscopic Roux-en-Y gastric bypass (LRYGB). Little large-volume data exist on how to handle the mesenteric defects during LRYGB. This study evaluated long-term follow-up (5.5 years) of 2443 patients with primary closure of the mesenteric defects with a stapling device at LRYGB, in comparison with a non-closed group from the same centre.

**Methods:**

All patients (*N* = 4013) undergoing LRYGB over a 10-year period (2005–2015) at a single institution were evaluated. The mesenteric defects were routinely closed starting June 2010. In total, 1570 non-closure patients and 2443 patients with stapled closure of the defects were prospectively entered and the results analysed.

**Results:**

Closure of the mesenteric defects increased surgical time by 4 min and did not affect the 30-day complication rate. IH incidence was significantly lower (2.5%) in the closure group compared with 11.7% in the non-closure group, at 60 months. The relative risk reduction by closing the mesenteric defects was 4.09-fold (95% CI = 2.97–5.62) as calculated using a survival model.

**Conclusions:**

Internal hernia after LRYGB occurs frequently if mesenteric defects are left unclosed. Primary closure with a hernia-stapling device is safe and significantly reduces the risk of internal hernia.

**Electronic supplementary material:**

The online version of this article (doi:10.1007/s00464-017-5415-2) contains supplementary material, which is available to authorized users.

Laparoscopic Roux-en-Y gastric bypass (LRYGB) is one of the most commonly performed bariatric procedures. In the USA alone, more than 50000 LRYGBs are performed each year accounting for approximately 45% of all bariatric procedures [1]. LRYGB is even more widely used in the Nordic countries and presently accounts for 82% of all bariatric surgery in the Scandinavian Obesity Surgery Registry SOReg [2].

IH remains a major cause of late complications with a reported incidence of 0.5–11% [3–7] and is thought to be higher in patients undergoing a laparoscopic versus an open approach. Due to lack of other reliable diagnostic modalities and the potentially catastrophic risk of a missed or delayed diagnosis [7], laparoscopic exploration remains essential in suspected cases. Consequently, pre-emptive measures are of great importance. Two systematic reviews [8, 9] have highlighted the low quality of included studies (level 3 or 4), with varying IH definitions, lack of information on closure techniques, and insufficient follow-up. According to one meta-analysis, the lowest incidence of IH was in the antecolic group with suture closure of both defects followed by the antecolic group with all defects left open [8]. Many advocate primary closure of mesenteric defects, and non-absorbable suture is often recommended [4, 6], but no consensus has been reached on this topic. In 2010, a new method of closing the mesenteric defects was introduced using a stapler device (Endohernia**®** stapler) [10]. As this method of closure has been implemented in many bariatric centres throughout Scandinavia and in other European countries, it seems important to report the 5-year results on the efficacy and safety of this method, from our high-volume centre.

## Materials and methods

The present study is a longitudinal cohort study, with a historic control patient material; all data for both groups were prospectively collected.

### Patient material

Aleris Hospital Oslo is a high-volume centre with a dedicated bariatric unit, presently performing around 800 bariatric procedures a year. All consecutive LRYGBs performed between 2005 and November 2015 were included in the study. Indications for surgery were in line with the European guidelines on surgery of severe obesity [11]. Informed consent was obtained from all individual participants included in the study.

### Operative procedure

The surgical procedure has been described in detail previously [12]. In brief, a small gastric pouch (15 mL) is created, and the jejunum brought up, first as an “omega” loop in an antecolic and antegastric fashion. Following linear stapling of the anastomoses and division of the omega loop, the last step is testing the integrity of the gastroenterostomy by inflating it with methylene blue-dyed saline via a temporary NG-tube. Routine limb lengths were 150 cm for the alimentary limb and 60 cm for the bilio-pancreatic, except that most patients with BMI > 48 Kg/m^2^ underwent operation with a 2 m bilio-pancreatic limb. All surgeons participating had already at the start of the study performed at least 1000 LRYGB operations.

### Previous policy

During the period from 2005 until May 2010, we performed a total of 1570 LRYGBs without closing the mesenteric defects.

### New policy

The surgical community became increasingly aware of the problem of internal herniation. So, as of 1 June 2010 to 1 November 2015, we have performed 2443 LRYGBs and these with the mesenteric defects stapled as described previously [10].

### Closing mesenteric defects

**Fig. 1:**
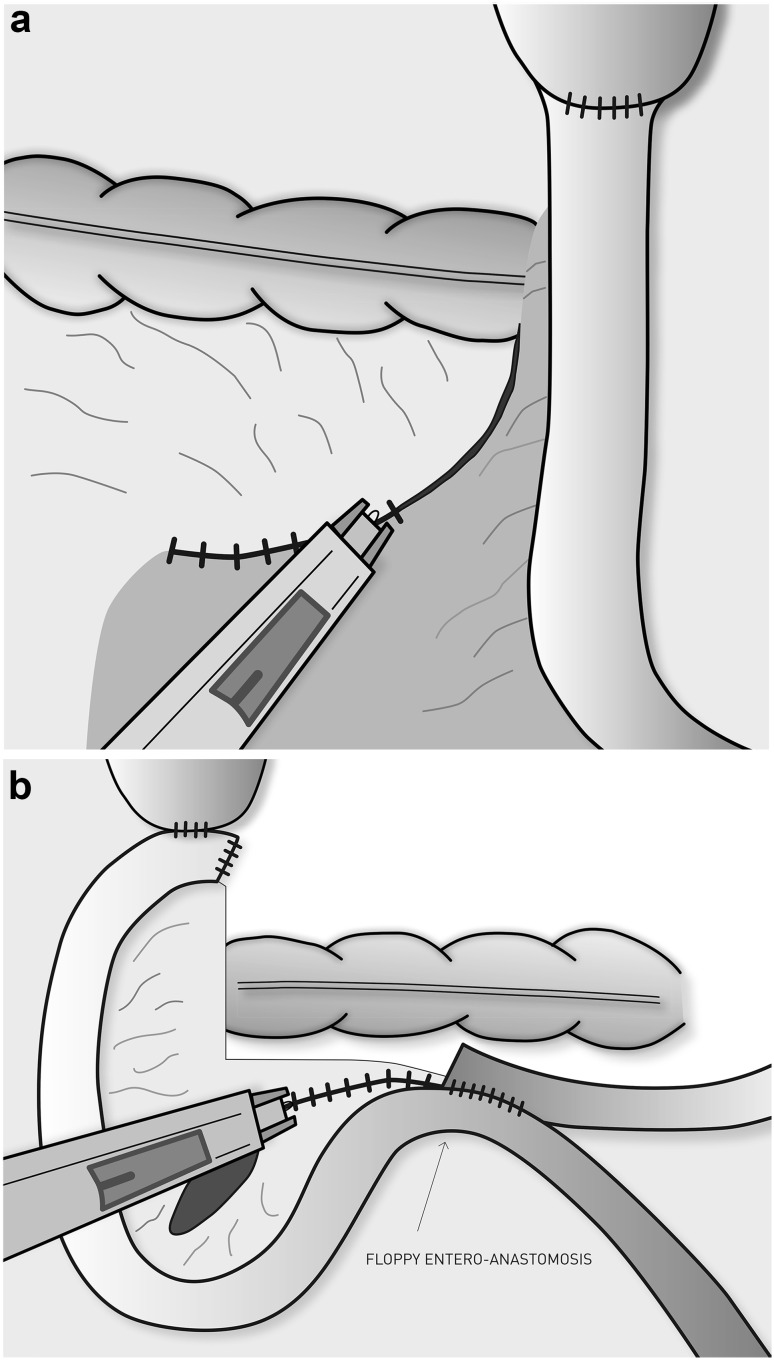
**A** Closure of Petersen’s space **B** Closure of jejunal mesenteric defect

The closing procedure is started after division of the omega loop and of the mesentery close to the gastroenterostomy. We now carry this division down to the edge of the transverse colon, usually about 5 cm down including the marginal vessels. Thus, the enteroenterostomy (EE) will be lying mobile below the transverse colon. The Endohernia® stapler (Endo Universal^TM^ 4.8 mm stapler, Autosuture) is inserted via a 12-mm port in the left upper abdomen. With gentle manoeuvring, graspers are used to expose the subcolic space behind the alimentary arm (“Petersen’s defect”) by lifting the transverse colon (Fig. 1A). The staples are partially extended presenting “hooks” that facilitate the catching and adaptation of the mesenteric peritoneum. Great care was taken to avoid deep bites in order to avoid damage to mesenteric vessels. The Petersen’s defect was closed from the root of the mesentery of the Roux limb and transverse mesocolon up to the transverse colon itself. To close the jejunal mesenteric defect, the assistant grasps the end of the duodenal limb and lifts the EE thereby exposing the mesenteric defect behind the EE (Fig. 1B). The same port and stapler are used for the closing of this defect. The details of the closure technique are demonstrated in the 4-minute video supplied (see Video, Supplemental Digital Content 1).

### Definition of internal hernia

All mesenterial openings in the present study were diagnosed by means of laparoscopy, and classified into two groups:

1. Internal hernia, defined as the presence of herniated small bowel with or without obstruction or ischemia through one or both mesenteric defects. Incidental IHs, found at laparoscopies conducted for uncertain abdominal pain, were included as having IH.
2. Suspected intermittent IH defined as clinical suspicion of IH and/or signs on CT scan, but at laparoscopy presenting open mesenteric defects without intestinal loop. Remission of complaints after closure of the mesenteric defects at three months follow-up were interpreted as confirmation of suspected intermittent IH.

### Data collection and statistics

Data were prospectively collected and registered in our database as part of our patient records. All data, including weight loss, metabolic status, and postoperative changes as well as complications, were registered continuously. All patients operated at our department were carefully instructed that abdominal symptoms possibly related to the surgery are taken care of at our department, including diagnostics and intervention, without any extra charge. Long-term follow-up (5 years and more after surgery) for complications and metabolic assessment was achieved in 71% (2851/4013) patients.

Statistical analyses were performed using SPSS for MacOS, version 22.0. Values are reported as median and range (or 95% confidence interval) unless otherwise stated. Time to reoperation for IH was estimated with the Kaplan–Meier method. All patients were followed up to first reoperation for IH and thereafter excluded from analysis if lost to follow-up, death or if mesenteric defects were closed at any reoperation. The relative risk reduction was estimated using Cox’s proportional hazards regression model without adjustment for other factors.

## Results

Between 2005 and 2015, 4013 patients underwent LRYGB at our institution. Of these, 1570 patients did not have their mesenterial openings closed (non-closure group, operated from 2005 to May 2010), and 2443 had both defects closed (operated from June 2010 to November 2015). There were no significant differences between the groups with respect to demographic data (Table 1).

**Table 1:** Demographic data at the time of LRYGB and postoperative complications

	2005–May 2010 non-closure	June 2010– November 2015 closure
Total number of patients	1570	2443
Age at operation, years	41 (18–72)	42 (17–76)
BMI at operation	42 (31–66)	40 (30–81)
FU time, months	77 (0–121)	40 (0–66)
Total number reoperations	279	67
Total number IH at operation	270	60
of which intermittent IH	85	10
IH site (% within IH positive group)		
Petersen	80 (43%)	26 (52%)
Jejunal mesenteric	72 (39%)	21 (42%)
Both	33 (18%)	3 (6%)
Complications to index operation		
Leakage	14 (0.9%)	18 (0.7%)
Bleeding Post-closure obstruction at EE	15 (0.9%) 0	24 (1.0%) 5

**Fig. 2:**
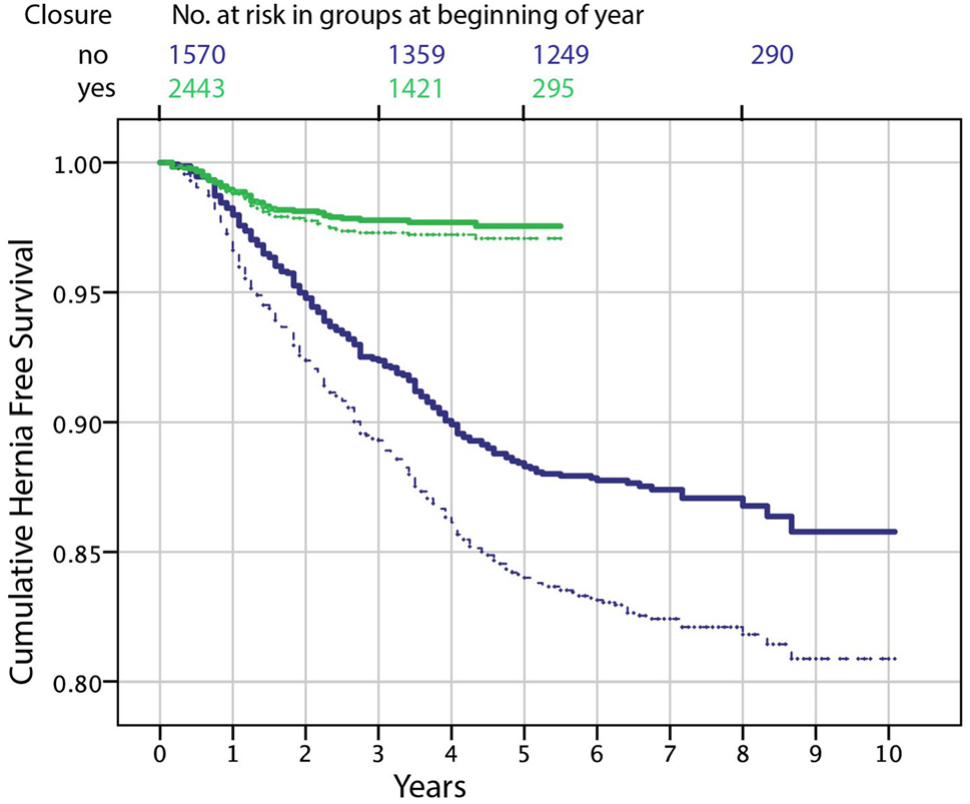
Survival function diagram using the Kaplan–Meier method demonstrating the relation of IH occurrence to post-operative time. The analysis is based on 4013 patients operated from 2005 to November 2015; number of patients at risk given at *top. Green* Closed mesenterial openings at index operation. *Blue* No closure of mesenterial openings at index operation. *Solid lines* Hernia containing bowel. *Dashed lines* Suspected hernia without bowel in mesenterial opening at laparoscopic exploration. Longer follow-up in the non-closure group as it preceded the closure group still allows a comparison at 5 years. (Color figure online)

The median hospital stay was 2 days (range 2–10). With a median follow-up of 77 months (0–121), 270 patients (17.2%) in the non-closure group developed a symptom of IH, requiring surgical intervention. Maximum follow-up in the closure group was 66 months, and in the non-closure group 121 months. When analysing data at 60-month FU time with a Kaplan–Meier estimate, the incidence of confirmed postoperative IH was significantly lower, 2.5% in the closure group compared to 11.7% in the non-closure group (Fig. 2).

The most frequent site of IH, Petersen’s space, was identified in 80 patients in the non-closure group and 26 in the closure group (Table 1).

There were signs of impaired alimentary limb emptying (kinking) in five of the first 40 patients in the closure group. With a change of surgical technique, viz. the division of the jejunal mesentery for a distance of 4–5 cm, these problems disappeared. There was no significant difference in kinking incidence when calculating between the entire two groups (chi square p = 0.0729).

Two hundred ninety-nine patients were readmitted to our hospital with clinical symptoms of IH. The diagnosis was further explored laparoscopically in all patients, and reduction of the hernia and/or closure of the mesenteric defects was undertaken with non-absorbable running sutures. No patient needed bowel resection. Six patients were pregnant; 4 in the first trimester and 2 in the third trimester. The remaining 31 patients were admitted acutely to other hospitals. Of them, fourteen were operated on with open surgery; seven patients needed bowel resections (50–300 cm) because of severe ischemia. One patient died at an outside hospital from septic complications associated with internal hernia.

The hernia-free survival function diagram (Fig. 2) illustrates how the IH cases in the non-closure group start to occur at about 2 months post-operatively and then seem to be uniformly distributed through the first 6 years. For comparison, the vast majority of the IH’s in the closure group (green lines) occurred within 3 years. No IH was registered in the closure group after >52 months, with 665 patients remaining at risk. The overall, unadjusted, relative risk reduction for confirmed IH was 4.09-fold (95% CI = 2.97–5.62) as estimated using Cox’s proportional hazards regression model comparing the rates of confirmed hernia in the two groups. Neither differences in limb length nor maximum EWL were found to be associated with increased risk of IH.

## Discussion

The overall medical benefits of gastric bypass are established, but the rate of adverse events has not been fully studied. Our cumulative incidence of intraoperatively verified IH of 11.8% (185/1571), and of 17.2% (270/1571) if suspected intermittent IH’s are included, is considerably higher than previously reported for LRYGB where the mesenteric defects were left open. Non-closure of the defects with the antegastric, antecolic LRYGB technique, as used in our material, have been reported to result in an IH-rate of 1–14.5% [3, 5, 13–17]. A lack of clear definition of IH and variations in follow-up might be responsible for the wide variation of incidence reported in the literature. In our series, the rate of IH is comparable to some other studies [4, 6, 16, 18] if only patients with demonstrated intestine in the mesenterial defect are included. However, we suggest that patients with clinical symptoms of IH that become asymptomatic after closure of the mesenterial defects should be included as suspected intermittent IH. Another important parameter influencing the incidence figure of IH is the long-term follow-up rate and the geographic considerations. In our practice, all patients were Norwegian citizens and therefore ended up either at our department in case of complications or were reported to us through national surveillance programmes. Therefore, the probability of missed cases is low. Long-term follow-up (5 years and more after surgery) for complications and metabolic assessment was achieved in 71% (2851/4013) patients.

Using the Kaplan–Meier technique, we found that our novel method of closure with the stapler resulted in an IH-rate of 2.5% (60/2444) over five years. This is in contrast to our observed five-year rate of 11.7% for patients left with the mesenterial openings intact. The reduction is highly significant as confirmed by using a Cox regression model; the overall reduction in relative risk is 4.086-fold with a significant confidence interval of 2.97–5.62. This result is not only comparable to, but probably better than what other authors have found using the closure technique with non-absorbable running sutures [19–21].

One aspect of closing mesenteric defects is time consumption and ease of use. Proper suturing of the defects is technically challenging compared to our method of stapling, especially in superobese patients. Suturing may also entail increased risk for mesenteric hematomas and anatomical disturbances causing secondary kinking and bowel obstruction [9, 19]. In the present study, there was no difference in complication rate between closure and non-closure groups, except in the first 40 patients where we saw several cases of kinking at the EE (Table 1) causing early obstruction or later discomfort and cramps. We found that a deeper division of the mesentery using the Ultrasonic shears as demonstrated in the video (Supplemental material) relieved this tendency without causing intestinal ischemia or GE-stricture. The rate of kinking in the present study was however not statistically significant when analysing the entire two groups of patients. The effect of deeper mesenterial division was immediately evident in our high-volume practice and was simultaneously observed at another high-volume centre where the same technique change was introduced. We therefore consider it unlikely that this effect depends on other factors and that it would be ethically questionable to conduct a randomized controlled trial. Leakage and haemorrhage occurred in 1.7% overall, which is on par with some of the lowest complication rates in published reports [22].

Suture material for mesenteric closure is less costly than staples, but we estimate that this difference is ameliorated in our high-volume, fast-track setting by less time consumption.

By dividing the small bowel mesentery thoroughly, at least to below the upper edge of the transverse colon, the EE becomes mobile, resulting in easier emptying of both the alimentary limb and the bilio-pancreatic limb. Other techniques such as extra stiches to align the lower end of the alimentary limb to the EE or a long EE (double-stapled) have been suggested to give the same advantage but this remains unconfirmed.

Our method is technically unchallenging (see Video, Supplemental Digital Content 1) and expeditious, with only 4 min added to total operating time. It can be applied in all patients without affecting the complication rate. Other techniques for the closure of mesenteric defects at the time of primary surgery such as mesenteric abrasion and fibrin glue have been described [23, 24]. In an experimental study, we found no benefits to these techniques as compared to stapling [25].

It seems that the laparoscopic approach is associated with a higher risk of internal hernias than that of open gastric bypass, probably due to less postoperative adhesions [26]. The clinical presentation of IH ranges from intermittent pain, often in the left upper abdomen to more constant abdominal pain, with or without nausea and vomiting to severe, acute abdominal pain [10, 17, 27]. A positive diagnosis of IH can most often only be made by laparoscopy. Some IH seem to reduce spontaneously, with intermittent attacks of pain as the presenting symptom. Patients with intermittent symptoms but unconfirmed hernia at closure were followed up with a telephone interview three months later to evaluate the outcome.

In case of uncertain abdominal pain in patients with LRYGB, our policy is to perform gastroscopy to exclude ulcers and ultrasound examination gallstones. Whenever a laparoscopy is indicated in these patients, whether diagnostic or for cholecystectomy, it is mandatory in our practice to close the mesenterial defects.

Closure patency is dependent on several factors, such as surgical skill, the technique used as well as on patient factors. The defects may reopen, permitting IH despite primary closure. In our study, like in many others [4, 7, 14, 15, 21] primary closure significantly reduced but did not eliminate the risk of IH. Since all surgeons participating in the study had previously performed in excess of 1000 LRYGB each, the risk of learning curve effects could virtually be disregarded. Demographic data for the two groups did not differ, so any differences in outcome between the two groups is most probably due to the change in technique.

A recent multicentre, randomized clinical trial of 2507 patients revealed a lower IH incidence rate in the routine closure group within a 2-year time frame [19]. They used the same technique as we do, known as the Lönroth technique [28]. In the original technique, the small mesenteric window at the level of dividing the omega loop was thought to be an advantage in preventing IH. However, closure of the mesenteric defects proves to be superior to the unclosed mesenteric defects. This study also highlighted the risk of kinking at the EE due to fixation by the mesenterial closure technique. A floppy EE accomplished by partial division of the mesentery seems to prevent this severe early complication. We have demonstrated in animal experiments that closure with non-absorbable sutures and clips both result in high tensile strength [25]. As a team policy, we have done all secondary closure of mesenteric defects with running non-absorbable sutures. However, in light of our results closure with the stapling device, it could be considered also in secondary procedures. Future comparative studies will add important information on the best method for closure.

## Conclusion

Our experience is in line with increasing evidence from several centres that the mesenteric defects should be closed primarily. Our method of primary closure with Endohernia**®** stapling is safe and results in a significantly reduced risk of internal hernia. Regardless of whether the mesenteric defects in LRYGB were primarily closed, suspicion of IH in patients with acute or chronic abdominal pain is still mandatory.

## Electronic supplementary material

Below is the link to the electronic supplementary material.

Supplementary material 1 Video that demonstrates the closure technique of the mesenteric defects in LRYGB with a stapling device (MOV 275767 KB)
